# Supplementary material for: Psychological, social and cognitive resources and the mental wellbeing of the poor
Source: PLoS One. 2021 Oct 12;16(10):e0258417. doi: 10.1371/journal.pone.0258417 (PMC8509876; doi:10.1371/journal.pone.0258417)
Supplement: S1 Online Appendix — (DOCX) [file pone.0258417.s001.docx]

**Online Appendix**

# Appendix S1: The Data

## A.1. Journeys Home

Journeys Home is a longitudinal survey of Australians who were either homeless or at risk of homelessness when first surveyed. The survey began in September 2011 and concluded in May 2014. It consists of six bi-annual waves of data. Prospective participants were identified from an administrative dataset drawn from the customer database from Australia’s central welfare agency (Centrelink). Centrelink’s database contains an indicator for people who have been flagged as ‘homeless’ or ‘at risk of homelessness’, which is determined using Centrelink’s Homelessness Indicator service delivery tool. In flagging homelessness, Centrelink takes the following definition into account (Wooden et al. 2012):

A person who is ‘homeless’ is one that:

- is without conventional accommodation (e.g., sleeping rough, squatting or living in a car); or
- lives in, or moves frequently between, temporary accommodation arrangements (e.g., with friends or extended family, emergency accommodation, or youth refuges).

A person who is ‘at risk’ of homelessness is one that:

- lives medium to long term in a boarding house, caravan park or hotel, where accommodation is not covered by a lease;
- lives in accommodation which falls below the general community standards which surround health and well-being, such as access to personal amenities, security against threat, privacy, and autonomy;
- is facing eviction; or
- lives in accommodation not of an appropriate standard, which may be detrimental to their physical and mental well-being, or where they have no sense of belonging or connection (e.g., Indigenous Australians living in crowded conditions or disconnected from their land, family/ kin, spiritual, and cultural beliefs and practices).

The sampling strategy also included identifying people who had not been flagged by Centrelink’s homelessness indicator but were otherwise statistically similar to those who had been flagged. 1,682 people aged 15 and older participated in the first wave of Journeys Home. Across the six waves, attrition was relatively low, with participation always remaining above 80% of those surveyed in wave 1 (see Fig A1).

**Fig A1. Sample Participation Across Six Waves of Journeys Home**

## A.2. Journeys Home Analysis Sample

We restrict the analysis sample to people who actually experienced homelessness during the Journeys Home survey period. This ensures that we have a more homogenous group with a well-defined definition of disadvantage. In practice, 80% of people reported an episode of homelessness at least once during the study period. This leaves us with a sample of 1,337 individuals before imposing age restrictions (we retain only those aged 18 years and older) and before accounting for item or wave non-response. We primarily use data from waves 5 and 6 when information on psychological resources was collected. The exact sample we use varies depending on which psychological resources we are interested in. This is discussed later when we analyze the gaps in psychological resources between our Journeys Home and Australian population samples.

For the purpose of restricting our sample, we adopt the Melbourne Institute definition of homelessness which categorizes people into different levels of housing vulnerability (Bevitt et al. 2014). Specifically, the Melbourne Institute categories follow the cultural definition of homelessness that assesses whether people’s accommodation meets the minimum community standard that people can expect to achieve in contemporary Australian society (Chamberlain & Mackenzie 1992). The labels used by the Melbourne Institute are in square brackets.

- ***Homeless*** *[Primary homeless]* captures anyone sleeping rough, squatting, or living in a car not by their own choice. People living like this are considered to be without housing.

Those with housing – who nonetheless are considered to be homeless – include those living/staying i) with friends, family, or other households; ii) in a boarding house/motel/hostel/hotel; iii) in accommodation provided by welfare services; iv) in a caravan; or v) in crisis accommodation, who are:

- ***Insecure non-traditional housing*** *[Secondary homeless]*, which captures those in this situation temporarily (which is defined as ‘having stayed in current place for 3 months or less and cannot stay there for the next 3 months’) and/or not sleeping in a bedroom; or
- ***Secure non-traditional housing*** *[Tertiary homeless]*, which captures those in this situation long-term and living in a caravan/boarding house or housing provided by welfare services or crisis accommodation.

We classify as homeless those who either meet the homeless, insecure non-traditional housing or secure non-traditional housing definition of homelessness at interview or spent any time between interviews living:

- In the primary homeless categories; or
- In a caravan, hotel, boarding house or in crisis accommodation; or
- With friends, family, or other relatives but not for more than 6 months.

For the first wave, we also include people who report homelessness in the six months preceding their interview.

## A.3. HILDA Survey

The Household Income and Labour Dynamics in Australia (HILDA) survey is a representative longitudinal survey that was launched in 2001 and has followed a panel of Australian households annually since. It began with 19,914 individuals belonging to 7,682 households. In 2011 the sample was replenished with an additional 5,462 people belonging to 2,153 households. The wave-on-wave response rate has generally been high at around 95% most years (Summerfield et al. 2019).

## A.4. Main Variables

Our objective is to capture the qualities, skills and support systems that help people achieve their goals and deal with problems effectively. We focus on the notion of “resources” as an umbrella term to capture existing concepts across psychology, economics, sociology, and cognitive sciences that support wellbeing and positive life outcomes. In Table A1, we outline how each key resource is measured in the respective surveys, how we reconstruct them to achieve comparability, and, where applicable, the ordinal alpha values.

**Table A1: Main Variables**

| **Variable** | **In JH** | **In HILDA** | **Notes** |
| --- | --- | --- | --- |
| Emotional stability | W5 (2013) | W13 (2013) | Big-5 personalities traits were elicited using the trait descriptive adjectives approach of Saucier (1994). In HILDA, item responses are from agreement regarding personal characteristic traits using a 7-point scale, while Journeys Home uses a 5-point scale. To achieve comparability, we convert scores in both surveys to 3-point scales (i.e. disagree, neutral, agree). Emotional stability is the summation of agreement to the following: *moody* (reversed), *touchy* (reversed), *temperamental* (reversed). HILDA also includes *envious*, *jealous,* and *fretful*: however, we ignore these because they are not in Journeys Home. Our modified emotional stability scale has ordinal alpha = 0.75. |
| Conscientiousness | W5 (2013) | W13 (2013) | See emotional stability for further details. Conscientiousness is the summation of agreement to the following: *orderly*, *sloppy* (reversed), *disorganised* (reversed), *efficient*. HILDA also includes *systematic* and *inefficient*; however, we ignore these because they are not in Journeys Home. Our modified conscientiousness scale has ordinal alpha = 0.79. |
| Cognition | W6 (2014) | W12 (2012) | Backwards Digits span score (0-8). Participants in both surveys were asked to recall increasing number of digits, read out by the interviewer, in reverse order as a generic test of working memory. |
| Internal locus of control | W6 (2014) | W15 (2015) | Questions on personal control are adapted from Pearlin and Schooler (1978). In HILDA, item responses are from agreement regarding personal control tendencies using a 7-point scale, while Journeys Home uses a 5-point scale. To achieve comparability, we convert scores in both surveys to 3-point scales (i.e. disagree, neutral, agree). The externality score is the summation of seven items: *You have little control over the things that happen to you* (reversed); *There is really no way you can solve some of the problems you have* (reversed); *There is little you can do to change many of the important things in your life* (reversed); *You often feel helpless in dealing with the problems of life* (reversed); *Sometimes you feel that you are being pushed around in life* (reversed); *What happens in the future mostly depends on you*; *You can do just about anything you really set your mind to do*. Our modified external locus of control scale has ordinal alpha = 0.88. |
| Risk willingness | W6 (2014) | W14 (2014) | Stated willingness to take risks in general (see e.g. Dohmen et al. 2011), with minor language discrepancies. In Journeys Home, the question is: “On a scale of 0 to 10, where 0 means you are unwilling to take any risks and 10 means you are very willing to take risks, how willing or unwilling to take risks would you say you are?” In HILDA the question is: “Are you generally a person who is willing to take risks or are you unwilling to take risks? Please indicate by crossing one box below. The more willing you are to take risks the higher the number of the box you should cross. The less willing you are to take risks, the lower the number of the box you should cross.” |
| Social capital | W6 (2014) | W14 (2014) | Social capital (support) questions are based on Henderson et al. (1978) and Marshall and Barnett (1993). We use the four overlapping social support questions from the 10 contained in HILDA (excluding the question on loneliness, which we use as a separate construct). In HILDA, item responses are from agreement regarding feelings using a 7‑point scale, while Journeys Home uses a 5-point scale. To achieve comparability, we convert scores in both surveys to 3‑point scales (i.e. disagree, neutral, agree). The social capital score is the summation of the following items (in Journeys Home, ‘I’ is replaced by ‘you’ etc.): *I often need help from other people but can’t get it?* (reversed); *I have no one to lean on in times of trouble* (reversed; in Journeys Home this is asked in reverse i.e. ‘You have someone you can lean on in times of trouble’); *There is someone who can always cheer me up when I’m down*; *When something’s on my mind, just talking with the people I know can make me feel better*. Our social capital scale has ordinal alpha = 0.73. |
| Sleep quality | W6 (2014) | W13 (2013) | Self-assessed sleep quality based on the question: *During the past month, how would you rate your sleep quality overall?* (answers coded as: very good (4); fairly good (3); fairly bad (2); very bad (1)). |
| Mental distress | W6 (2014) | W13 (2013) | Mental distress is measured using the K-6 mental distress questionnaire (Kessler et al. 2002), which is a short-form of the longer K-10. Although HILDA includes the full K-10, Journeys Home only includes the K-6. Participants were asked how often they had experienced the following in the past four weeks: *how often did you feel nervous?*; *how often did you feel without hope?*; *how often did you feel restless or fidgety?*; *how often did you feel that everything was an effort?*; *how often did you feel so sad that nothing could cheer you up?*; *how often did you feel worthless?*. Answers are on a 5-point scale: all of the time (0); most of the time (1); some of the time (2); a little of the time (3); none of the time (4). The K-6 score is the summation of the reversed values for each of these items. This score has ordinal alpha = 0.94. |
| High mental distress | W6 (2014) | W13 (2013) | This corresponds to a K-6 score of ≥ 13 points, which has been found to be optimal for screening for serious mental illness (Kessler et al. 2003). |
| Life satisfaction | W6 (2014) | W13 (2014) | A global reflection of overall life satisfaction captured by the question: *All things considered, how satisfied are you with your life?* (answers coded on an 11-points scale: totally dissatisfied (0) … totally satisfied (10)). |
| Loneliness | W6 (2014) | W14 (2014) | This is agreement to the statement: *I often feel very lonely* (in Journeys Home, ‘I’ is replaced by ‘you’). In HILDA, degree of agreement uses a 7-point scale, while Journeys Home uses a 5-point scale. To achieve comparability, we convert scores in both surveys to an indicator for being lonely (i.e. do not agree (0), agree (1)). We use an indicator because there are few people in the neutral category and this eases interpretation. |

Notes: In Journeys Home, Wave 5 was conducted between September‑November 2013 and Wave 6 between March-May 2014. HILDA interviews are primarily in August‑October of each year.

## A.5. Other Variables

We identified several common demographic and family background variables that we use as controls. These are described in Table A2.

**Table A2: Other Variables**

| **Variable** | **Definition** |
| --- | --- |
| Age | Age in years |
| Male | =1 if male |
| ATSI | =1 if Aboriginal or Torres Strait Islander |
| Student | =1 if studying full-time |
| University | =1 if highest education achieved is bachelor’s degree or higher (e.g. postgraduate degree, graduate diploma) |
| Diploma | =1 if highest education achieved is diploma |
| Certificate 3 or 4 | =1 if highest education achieved is certificate 3 or 4 |
| Year 12 | =1 if highest education achieved is year 12 |
| Married | =1 if current marital status is in a registered marriage |
| Defacto | =1 if current marital status is in a defacto relationship |
| Separated | =1 if current marital status is separated |
| Divorced | =1 if current marital status is divorced |
| Single | =1 if current marital status is single |
| Aus. Born | =1 if born in Australia |
| Born main English | =1 if born in other main English speaking country |
| Long term health cond.^a^ | =1 if has a long-term health condition |
| Parents separated age 16 | =1 if parents were separated at any point before age 16 but were together at birth |
| Parents never together | =1 if parents were never together |
| Mother university degree | =1 if mother has a university degree |
| Major urban^b^ | =1 if lives in a major urban area |
| Other urban^b^ | =1 if lives in an other urban area |
| Rural balance^b^ | =1 if lives in rural balance |

Notes: ^a^HILDA participants received a showcard listing examples of health conditions while Journeys Home participants did not. ^b^Based on the Australian Bureau of Statistics’ Australian Statistical Geography Standard classifications. Baseline is ‘bounded locality’, which is between ‘other urban’ and ‘rural balance’ in urbanicity.

**Appendix S1 References:**

Bevitt A, Chigavazira A, Scutella R, Tseng YP, Watson N. Journeys Home User Manual, User Manual Version: 6.0.2, Data Release Version: 201412.1. Melbourne: Melbourne Institute of Applied Economic and Social Research, University of Melbourne; 2014.

Chamberlain C, Mackenzie D. Understanding contemporary homelessness: Issues of definition and meaning. Aust J Soc Issues. 1992;27(4):274–297.

Dohmen T, Falk A, Huffman D, Sunde U, Schupp J, Wagner GG. Individual risk attitudes: Measurement, determinants, and behavioral consequences. J Eur Econ Assoc. 2011;9(3):522–550.

Henderson S, Duncan-Jones P, McAuley H, Ritchie K. The patient’s primary group. Br J Psychiatry. 1978;132(1):74–86.

Kessler RC, Andrews G, Colpe LJ, Hiripi E, Mroczek DK, Normand SL, Walters EE, Zaslavsky AM. Short screening scales to monitor population prevalences and trends in non-specific psychological distress. Psychol Med. 2002;32(6):959–976.

Kessler RC, Barker PR, Colpe LJ, Epstein JF, Gfroerer JC, Hiripi E, Howes MJ, Normand SL, Manderscheid RW, Walters EE, Zaslavsky AM. Screening for serious mental illness in the general population. JAMA Psychiatry. 2003;60(2):184–189.

Marshall NL, Barnett, RC. Work‐family strains and gains among two‐earner couples. J Community Psychol. 1993;21(1):64–78.

Pearlin LI, Schooler C. The Structure of coping. J Health Soc Behav. 1978;19(1):2–21.

Saucier G. Mini-Markers: A brief version of Goldberg's unipolar Big-Five markers. J Pers Assess. 1994;63(3):506–516.

Summerfield M, Bright S, Hahn M, La N, Macalalad N, Watson N, Wilkins R, Wooden M. HILDA User Manual – Release 18. Melbourne: Melbourne Institute of Applied Economic and Social Research, University of Melbourne; 2019.

Wooden M, Bevitt A, Chigavazira A, Greer N, Johnson G, Killackey E, Moschion J, Scutella R, Tseng YP, Watson N. Introducing ‘Journeys Home’. Aust Econ Rev. 2012;45(3):368–378.

# Appendix S2: Comparing Psychological, Social and Cognitive Resources Across Populations

**Table B1: Unconditional Mean Differences in Psychological Resources**

| **Resource** | **Journeys Home sample size** | **HILDA sample size** | **Mean difference (SE)** | |
| --- | --- | --- | --- | --- |
| Conscientiousness | 1,000 | 14,208 | | -0.44 (0.050) |
| Emotional stability | 1,070 | 14,231 | | -0.49 (0.043) |
| Cognition | 1,077 | 15,540 | | -0.32 (0.044) |
| Internal locus of control | 1,105 | 14,397 | | -0.50 (0.050) |
| Risk willingness | 1,112 | 14,523 | | 0.57 (0.044) |
| Social capital | 1,117 | 14,419 | | -0.09 (0.046) |
| Sleep quality | 1,120 | 14,261 | | -0.29 (0.050) |

**Appendix S3: Accounting for Sample Overlap**

One concern with comparing Journeys Home and HILDA data is that the populations overlap. In practice, the severely disadvantaged subpopulation (captured by housing vulnerability) make up a small fraction of the general Australian population, making it reasonable to treat our samples as mutually exclusive groups. Nevertheless, an arguably cleaner comparison would be between those who are severely disadvantaged and those not severely disadvantaged (i.e., those in HILDA who do not also belong to the Journeys Home population). One way to make this comparison is to weight the HILDA sample by the probability that they actually are not disadvantaged.

To create weights, we estimate a scaled binomial loss model (Phillips & Elith 2011; 2013) for the probability of belonging to the disadvantaged population. This model was developed for studies in ecology where the presence of a species is unknown in some background sample, but its probability of being present across the entire landscape is known.

Let $G$ denote the Australian general population (which HILDA is drawn from), which is comprised of the population of severely disadvantaged $G_{1}$ (which Journeys Home is drawn from) and the not-disadvantaged $G_{0}$. It is assumed that each sample (Journeys Home and HILDA) is drawn uniformly from $G_{1}$ with probability $f_{p}$ and from $G$ with probability $1-f_{p}$. $f_{p}$ is then $\frac{n_{jh}}{n_{jh}+n_{hilda}}$ where $n$ means sample size. The unconditional probability that person $i\in G$ also belongs to $G_{1}$ is denoted by $\pi$. The probability that person $i$ belongs to the Journeys Home sample can be expressed as:

$$\begin{aligned} P_{i}(jh=1|\boldsymbol{X}_{\boldsymbol{i}}\boldsymbol{)}=\frac{1}{1+r+\exp(-\boldsymbol{X}_{\boldsymbol{i}}^{\boldsymbol{'}}\boldsymbol{\delta}+\ln\left( r \right))}\#\left( 2 \right) \end{aligned}$$

where

$$\begin{aligned} r=\frac{\left( 1-f_{p} \right)\pi}{f_{p}}\#\left( 3 \right) \end{aligned}$$

The coefficients $\boldsymbol{\delta}$ are straightforward to estimate by maximizing:

$$\begin{aligned} LL=\sum_{i\in jh}^{n_{jh}} \ln P_{i}\left( jh=1 | \boldsymbol{X}_{\boldsymbol{i}} \right)\boldsymbol{+}\sum_{i\in hilda}^{n_{hilda}} \ln P_{i}\left( jh=1 | \boldsymbol{X}_{\boldsymbol{i}} \right)\#\left( 4 \right) \end{aligned}$$

A critical step is to specify a suitable value for $\pi$. In our case we can make a strongly informed estimate. From the Journeys Home user manual (Bevitt et al. 2014), we know that this sample was drawn from a population of 107,532 individuals aged 15 years and older. According to the Australian Census, there were 17,363,694 Australians aged 15 years and older in 2011. Noting that we only include the 80% of Journeys Home participants who actually experienced an episode of homelessness, we calculate $\pi=\frac{0.8\times107,532}{17,363,694}=0.004954337.$ That is, we expect 0.5% of those in HILDA to also belong to the severely disadvantaged population. We get a similar value for $\pi$ if we instead use the supplied population weights to infer the numerator.

After estimating Equation (2), we can convert these estimates into predicted probabilities for belonging to the severely disadvantaged population $(\tilde{Y_{i}}=1)$ using the fact that $P_{i}(\tilde{Y}_{i}=1|\boldsymbol{X}_{\boldsymbol{i}}\boldsymbol{)}$ is $r$ times the odds of $P_{i}\left( jh=1 | \boldsymbol{X}_{\boldsymbol{i}} \right)$ (Philips & Elith 2011);

$$\begin{aligned} P_{i}\left( \tilde{Y}_{i}=1 | \boldsymbol{X}_{\boldsymbol{i}} \right)\boldsymbol{=}r\frac{P_{i}\left( jh=1 | \boldsymbol{X}_{\boldsymbol{i}} \right)}{1\boldsymbol{-}P_{i}\left( jh=1 | \boldsymbol{X}_{\boldsymbol{i}} \right)}\#\left( 5 \right) \end{aligned}$$

We use $1-\hat{P}_{i}\left( \tilde{Y}_{i}=1 | \boldsymbol{X}_{\boldsymbol{i}} \right)$ to form weights for HILDA observations (in Fig 2 we rescale these weights so they sum to the HILDA sample size).

**Appendix S3 References:**

Bevitt A, Chigavazira A, Scutella R, Tseng YP, Watson N. Journeys Home User Manual, User Manual Version: 6.0.2, Data Release Version: 201412.1. Melbourne: Melbourne Institute of Applied Economic and Social Research, University of Melbourne; 2014.

Phillips SJ, Elith J. Logistic methods for resource selection functions and presence-only species distribution models. In: Proceedings of the Twenty-Fifth AAAI Conference on Artificial Intelligence; 2011 Aug 4; San Francisco, California. AAAI [Internet]; 2011. Available from <https://www.aaai.org/ocs/index.php/AAAI/AAAI11/paper/view/3657>.

Phillips SJ, Elith J. On estimating probability of presence from use–availability or presence–background data. Ecology*.* 2013;94(6):1409–1419.

# Appendix S4: Decomposition Analysis

Baseline decomposition results are reported in Table D1. These correspond to Fig 5 in the paper and are discussed in the main text.

**Table D1: Detailed Blinder-Oaxaca Decomposition Results – Baseline**

|  | Mental distress | High mental distress | Life satisfaction | Loneliness |
| --- | --- | --- | --- | --- |
| *Overall* |  |  |  |  |
| Mean (JH) | 0.840^***^ | 0.158^***^ | -0.579^***^ | 0.312^***^ |
|  | (0.068) | (0.017) | (0.074) | (0.022) |
| Mean (HILDA) | -0.066^***^ | 0.037^***^ | 0.021^*^ | 0.169^***^ |
|  | (0.012) | (0.003) | (0.012) | (0.005) |
| Difference | 0.906^***^ | 0.121^***^ | -0.600^***^ | 0.143^***^ |
|  | (0.069) | (0.017) | (0.075) | (0.023) |
| Explained | 0.525^***^ | 0.060^***^ | -0.355^***^ | 0.092^***^ |
|  | (0.037) | (0.007) | (0.034) | (0.013) |
| Unexplained | 0.382^***^ | 0.061^***^ | -0.245^***^ | 0.051^**^ |
|  | (0.057) | (0.018) | (0.068) | (0.021) |
| *Explained* |  |  |  |  |
| Risk willingness | -0.011 | -0.004^*^ | 0.007 | 0.003 |
|  | (0.007) | (0.002) | (0.008) | (0.003) |
| Internal locus of control | 0.109^***^ | 0.011^***^ | -0.085^***^ | 0.025^***^ |
|  | (0.014) | (0.002) | (0.012) | (0.004) |
| Cognition | 0.005 | 0.000 | 0.008^**^ | -0.001 |
|  | (0.003) | (0.001) | (0.004) | (0.001) |
| Emotional stability | 0.091^***^ | 0.008^***^ | -0.024^***^ | 0.014^***^ |
|  | (0.011) | (0.002) | (0.006) | (0.003) |
| Conscientiousness | 0.030^***^ | 0.005^***^ | -0.015^***^ | 0.000 |
|  | (0.006) | (0.002) | (0.005) | (0.002) |
| Social capital | 0.016^**^ | 0.002^**^ | -0.023^**^ | 0.014^**^ |
|  | (0.007) | (0.001) | (0.010) | (0.006) |
| Sleep quality | 0.057^***^ | 0.007^***^ | -0.044^***^ | 0.005^***^ |
|  | (0.012) | (0.002) | (0.009) | (0.002) |
| Long term health cond. | 0.022^***^ | 0.003^***^ | -0.019^***^ | 0.003^**^ |
|  | (0.005) | (0.001) | (0.005) | (0.001) |
| Parents separated age 16 | 0.009 | 0.002 | -0.008 | 0.005 |
|  | (0.008) | (0.002) | (0.009) | (0.004) |
| Parents never together | -0.005 | -0.001 | -0.003 | 0.001 |
|  | (0.005) | (0.001) | (0.007) | (0.003) |
| Mother university degree | -0.001 | -0.000 | 0.000 | 0.000 |
|  | (0.001) | (0.000) | (0.001) | (0.000) |
| Age | 0.115^***^ | 0.013^***^ | -0.109^***^ | 0.007 |
|  | (0.012) | (0.003) | (0.013) | (0.005) |
| Male | -0.016^***^ | -0.002^**^ | -0.008^**^ | -0.005^***^ |
|  | (0.004) | (0.001) | (0.004) | (0.002) |
| ATSI | 0.016^*^ | 0.003 | 0.009 | 0.003 |
|  | (0.009) | (0.003) | (0.011) | (0.005) |
| Student | 0.001 | -0.000 | 0.002 | -0.000 |
|  | (0.001) | (0.000) | (0.003) | (0.000) |
| University | -0.007 | 0.001 | 0.023^**^ | -0.005^*^ |
|  | (0.006) | (0.002) | (0.009) | (0.003) |
| Diploma | -0.000 | -0.000 | 0.001 | -0.000 |
|  | (0.001) | (0.000) | (0.002) | (0.000) |
| Certificate 3 or 4 | 0.001 | 0.000 | -0.006^*^ | 0.000 |
|  | (0.003) | (0.001) | (0.004) | (0.001) |
| Year 12 | -0.001 | 0.000 | 0.001 | -0.000 |
|  | (0.002) | (0.000) | (0.002) | (0.001) |
| Married | 0.037^*^ | 0.008 | -0.018 | 0.051^***^ |
|  | (0.021) | (0.006) | (0.028) | (0.012) |
| Defacto | 0.004 | -0.000 | 0.001 | -0.011^***^ |
|  | (0.005) | (0.001) | (0.006) | (0.003) |
| Separated | 0.005 | 0.001 | -0.016^***^ | -0.001 |
|  | (0.004) | (0.001) | (0.006) | (0.002) |
| Divorced | 0.004 | 0.000 | -0.009^**^ | -0.001 |
|  | (0.003) | (0.001) | (0.004) | (0.001) |
| Single | 0.043^**^ | 0.006 | -0.025 | -0.017^*^ |
|  | (0.020) | (0.006) | (0.023) | (0.010) |
| Aus. born | -0.003 | -0.000 | 0.012^**^ | 0.004^*^ |
|  | (0.005) | (0.002) | (0.006) | (0.002) |
| Born main English | 0.000 | -0.000 | -0.004 | -0.002^*^ |
|  | (0.002) | (0.001) | (0.003) | (0.001) |
| Major urban | -0.002 | -0.000 | 0.000 | 0.002 |
|  | (0.003) | (0.001) | (0.004) | (0.002) |
| Other urban | 0.002 | 0.000 | -0.002 | -0.001 |
|  | (0.002) | (0.000) | (0.002) | (0.001) |
| Rural balance | 0.003 | 0.000 | -0.003 | -0.001 |
|  | (0.002) | (0.001) | (0.003) | (0.001) |
| *Unexplained* |  |  |  |  |
| Risk willingness | -0.121 | -0.045 | 0.076 | -0.026 |
|  | (0.137) | (0.044) | (0.134) | (0.043) |
| Internal locus of control | -0.959^***^ | -0.266^***^ | 1.167^***^ | -0.327^***^ |
|  | (0.263) | (0.091) | (0.330) | (0.104) |
| Cognition | 0.011 | 0.001 | -0.403^**^ | 0.022 |
|  | (0.165) | (0.054) | (0.191) | (0.061) |
| Emotional stability | 0.237 | 0.006 | -0.058 | -0.025 |
|  | (0.161) | (0.053) | (0.189) | (0.061) |
| Conscientiousness | -0.110 | -0.008 | 0.464 | 0.048 |
|  | (0.227) | (0.073) | (0.283) | (0.081) |
| Social capital | -1.284^***^ | -0.389^***^ | 1.181^***^ | 0.017 |
|  | (0.300) | (0.106) | (0.384) | (0.125) |
| Sleep quality | -0.185 | -0.101^*^ | 0.381^**^ | -0.086 |
|  | (0.178) | (0.056) | (0.186) | (0.059) |
| Long term health cond. | 0.093^**^ | 0.010 | 0.019 | 0.021 |
|  | (0.042) | (0.013) | (0.049) | (0.016) |
| Parents separated age 16 | -0.022 | 0.004 | 0.020 | 0.012 |
|  | (0.052) | (0.016) | (0.063) | (0.018) |
| Parents never together | 0.013 | 0.001 | -0.005 | 0.003 |
|  | (0.011) | (0.004) | (0.018) | (0.006) |
| Mother university degree | 0.008 | -0.001 | -0.002 | 0.001 |
|  | (0.013) | (0.005) | (0.012) | (0.005) |
| Age | -0.145 | -0.033 | -0.321 | 0.044 |
|  | (0.170) | (0.052) | (0.212) | (0.065) |
| Male | 0.037 | 0.004 | -0.097 | 0.043^*^ |
|  | (0.067) | (0.021) | (0.074) | (0.025) |
| ATSI | -0.051^**^ | -0.011^*^ | 0.021 | -0.008 |
|  | (0.021) | (0.006) | (0.029) | (0.009) |
| Student | -0.015 | 0.004 | -0.020 | -0.001 |
|  | (0.011) | (0.004) | (0.013) | (0.004) |
| University | -0.010 | -0.003 | 0.003 | -0.000 |
|  | (0.009) | (0.002) | (0.007) | (0.004) |
| Diploma | 0.015 | -0.001 | -0.013 | 0.010 |
|  | (0.015) | (0.005) | (0.020) | (0.006) |
| Certificate 3 or 4 | -0.013 | -0.015 | -0.011 | 0.016 |
|  | (0.034) | (0.011) | (0.042) | (0.013) |
| Year 12 | 0.014 | 0.005 | -0.002 | 0.009 |
|  | (0.019) | (0.006) | (0.021) | (0.007) |
| Married | -0.048^*^ | -0.015^*^ | 0.011 | -0.014^**^ |
|  | (0.027) | (0.009) | (0.030) | (0.007) |
| Defacto | -0.171 | -0.073^*^ | 0.048 | -0.046 |
|  | (0.107) | (0.039) | (0.123) | (0.028) |
| Separated | -0.048 | -0.020 | 0.009 | -0.014 |
|  | (0.037) | (0.013) | (0.041) | (0.010) |
| Divorced | -0.059 | -0.031 | -0.008 | -0.010 |
|  | (0.054) | (0.020) | (0.062) | (0.013) |
| Single | -0.308 | -0.183^*^ | -0.072 | -0.082 |
|  | (0.284) | (0.104) | (0.332) | (0.073) |
| Aus. born | -0.023 | 0.024 | -0.456^**^ | -0.099 |
|  | (0.170) | (0.063) | (0.210) | (0.076) |
| Born main English | -0.009 | 0.003 | -0.042^*^ | -0.014^*^ |
|  | (0.017) | (0.006) | (0.023) | (0.008) |
| Major urban | 0.037 | 0.051 | -0.342 | -0.067 |
|  | (0.196) | (0.092) | (0.255) | (0.121) |
| Other urban | 0.008 | 0.007 | -0.050 | -0.016 |
|  | (0.043) | (0.019) | (0.055) | (0.025) |
| Rural balance | -0.004 | -0.001 | -0.030 | -0.002 |
|  | (0.012) | (0.005) | (0.020) | (0.008) |
| Constant | 3.495^***^ | 1.136^***^ | -1.715^**^ | 0.641^**^ |
|  | (0.726) | (0.259) | (0.823) | (0.250) |
| $n$ | 11612 | 11612 | 11695 | 11673 |
| $n_{jh}$ | 850 | 850 | 853 | 852 |
| $n_{hilda}$ | 10762 | 10762 | 10842 | 10821 |

Notes: See Section 3.2 for formulaic details. Estimates were obtained using the ‑oaxaca- command for Stata (Jann 2008). See Table A1 for further details on the dependent variables and psychological resource variables and Table A2 for further details on the other controls. Asymptotic standard errors in parentheses. * $p<0.1$, ** $p<0.05,$*** $p<0.01$.

We also performed decomposition using a weighted version of the equation for the HILDA sample as described in Appendix S3 (we multiply the sample overlap weights with population weights for the HILDA sample). Weighting this equation has only a minor effect on the estimates and does not change our conclusions (Fig D1 and Table D2).

**Fig D1.** **Gaps in Mental Wellbeing and Decomposition Results – Weighted for Population Group**

Notes: Graphical depiction of estimates in Table D2 (after normalizing to gap to equal one).

**Table D2: Detailed Blinder-Oaxaca Decomposition Results – Weighted for Population Group**

|  | Mental distress | High mental distress | Life satisfaction | Loneliness |
| --- | --- | --- | --- | --- |
| *Overall* |  |  |  |  |
| Mean (JH) | 0.840^***^ | 0.158^***^ | -0.579^***^ | 0.312^***^ |
|  | (0.068) | (0.017) | (0.074) | (0.022) |
| Mean (HILDA) | -0.070^***^ | 0.036^***^ | 0.023^**^ | 0.169^***^ |
|  | (0.012) | (0.003) | (0.012) | (0.005) |
| Difference | 0.910^***^ | 0.122^***^ | -0.602^***^ | 0.143^***^ |
|  | (0.069) | (0.017) | (0.075) | (0.023) |
| Explained | 0.526^***^ | 0.060^***^ | -0.356^***^ | 0.094^***^ |
|  | (0.037) | (0.007) | (0.034) | (0.013) |
| Unexplained | 0.384^***^ | 0.062^***^ | -0.246^***^ | 0.049^**^ |
|  | (0.057) | (0.018) | (0.068) | (0.021) |
| *Explained* |  |  |  |  |
| Risk willingness | -0.011^*^ | -0.004^*^ | 0.007 | 0.004 |
|  | (0.007) | (0.002) | (0.008) | (0.003) |
| Internal locus of control | 0.110^***^ | 0.011^***^ | -0.085^***^ | 0.025^***^ |
|  | (0.014) | (0.002) | (0.012) | (0.004) |
| Cognition | 0.005 | 0.000 | 0.008^**^ | -0.001 |
|  | (0.003) | (0.001) | (0.004) | (0.001) |
| Emotional stability | 0.092^***^ | 0.007^***^ | -0.025^***^ | 0.014^***^ |
|  | (0.011) | (0.002) | (0.006) | (0.003) |
| Conscientiousness | 0.030^***^ | 0.005^***^ | -0.015^***^ | 0.000 |
|  | (0.006) | (0.002) | (0.005) | (0.002) |
| Social capital | 0.016^**^ | 0.002^**^ | -0.023^**^ | 0.014^**^ |
|  | (0.007) | (0.001) | (0.010) | (0.006) |
| Sleep quality | 0.058^***^ | 0.007^***^ | -0.044^***^ | 0.005^***^ |
|  | (0.011) | (0.002) | (0.009) | (0.002) |
| Long term health cond. | 0.022^***^ | 0.003^***^ | -0.019^***^ | 0.003^**^ |
|  | (0.005) | (0.001) | (0.005) | (0.001) |
| Parents separated age 16 | 0.009 | 0.001 | -0.008 | 0.005 |
|  | (0.008) | (0.002) | (0.009) | (0.004) |
| Parents never together | -0.005 | -0.001 | -0.003 | 0.001 |
|  | (0.004) | (0.001) | (0.007) | (0.003) |
| Mother university degree | -0.001 | -0.000 | 0.001 | 0.000 |
|  | (0.001) | (0.000) | (0.001) | (0.000) |
| Age | 0.115^***^ | 0.013^***^ | -0.109^***^ | 0.007 |
|  | (0.012) | (0.003) | (0.013) | (0.005) |
| Male | -0.016^***^ | -0.002^**^ | -0.008^**^ | -0.005^***^ |
|  | (0.004) | (0.001) | (0.004) | (0.002) |
| ATSI | 0.015^*^ | 0.003 | 0.010 | 0.003 |
|  | (0.009) | (0.003) | (0.010) | (0.005) |
| Student | 0.001 | -0.000 | 0.002 | -0.000 |
|  | (0.001) | (0.000) | (0.003) | (0.000) |
| University | -0.006 | 0.000 | 0.023^**^ | -0.005^*^ |
|  | (0.006) | (0.001) | (0.009) | (0.003) |
| Diploma | -0.000 | -0.000 | 0.001 | 0.000 |
|  | (0.001) | (0.000) | (0.002) | (0.000) |
| Certificate 3 or 4 | 0.001 | 0.000 | -0.006 | 0.000 |
|  | (0.003) | (0.001) | (0.004) | (0.001) |
| Year 12 | -0.001 | 0.000 | 0.001 | -0.000 |
|  | (0.002) | (0.000) | (0.002) | (0.001) |
| Married | 0.037^*^ | 0.008 | -0.018 | 0.051^***^ |
|  | (0.022) | (0.006) | (0.029) | (0.012) |
| Defacto | 0.004 | -0.000 | 0.001 | -0.011^***^ |
|  | (0.005) | (0.001) | (0.006) | (0.003) |
| Separated | 0.005 | 0.001 | -0.016^***^ | -0.001 |
|  | (0.004) | (0.001) | (0.006) | (0.002) |
| Divorced | 0.004 | 0.000 | -0.009^**^ | -0.001 |
|  | (0.003) | (0.001) | (0.004) | (0.001) |
| Single | 0.043^**^ | 0.006 | -0.025 | -0.017^*^ |
|  | (0.021) | (0.006) | (0.023) | (0.010) |
| Aus. born | -0.003 | -0.000 | 0.012^**^ | 0.004^*^ |
|  | (0.005) | (0.002) | (0.006) | (0.002) |
| Born main English | 0.000 | -0.000 | -0.004 | -0.002^*^ |
|  | (0.002) | (0.001) | (0.003) | (0.001) |
| Major urban | -0.001 | -0.000 | -0.000 | 0.002 |
|  | (0.003) | (0.001) | (0.004) | (0.002) |
| Other urban | 0.002 | 0.000 | -0.002 | -0.001 |
|  | (0.002) | (0.000) | (0.002) | (0.001) |
| Rural balance | 0.002 | 0.000 | -0.003 | -0.001 |
|  | (0.002) | (0.001) | (0.003) | (0.001) |
| *Unexplained* |  |  |  |  |
| Risk willingness | -0.121 | -0.045 | 0.075 | -0.026 |
|  | (0.137) | (0.044) | (0.134) | (0.043) |
| Internal locus of control | -0.960^***^ | -0.267^***^ | 1.167^***^ | -0.327^***^ |
|  | (0.263) | (0.091) | (0.330) | (0.104) |
| Cognition | 0.011 | 0.001 | -0.405^**^ | 0.023 |
|  | (0.165) | (0.054) | (0.191) | (0.061) |
| Emotional stability | 0.235 | 0.005 | -0.059 | -0.025 |
|  | (0.161) | (0.053) | (0.189) | (0.061) |
| Conscientiousness | -0.112 | -0.009 | 0.466^*^ | 0.048 |
|  | (0.227) | (0.073) | (0.283) | (0.081) |
| Social capital | -1.284^***^ | -0.389^***^ | 1.182^***^ | 0.016 |
|  | (0.300) | (0.106) | (0.384) | (0.125) |
| Sleep quality | -0.189 | -0.102^*^ | 0.382^**^ | -0.087 |
|  | (0.178) | (0.056) | (0.186) | (0.059) |
| Long term health cond. | 0.093^**^ | 0.011 | 0.019 | 0.021 |
|  | (0.042) | (0.013) | (0.049) | (0.016) |
| Parents separated age 16 | -0.021 | 0.004 | 0.019 | 0.012 |
|  | (0.052) | (0.016) | (0.063) | (0.018) |
| Parents never together | 0.013 | 0.001 | -0.005 | 0.003 |
|  | (0.011) | (0.004) | (0.018) | (0.006) |
| Mother university degree | 0.008 | -0.001 | -0.002 | 0.001 |
|  | (0.013) | (0.005) | (0.012) | (0.005) |
| Age | -0.145 | -0.033 | -0.321 | 0.045 |
|  | (0.170) | (0.052) | (0.212) | (0.065) |
| Male | 0.037 | 0.004 | -0.097 | 0.043^*^ |
|  | (0.067) | (0.021) | (0.074) | (0.025) |
| ATSI | -0.050^**^ | -0.011^*^ | 0.020 | -0.008 |
|  | (0.021) | (0.006) | (0.028) | (0.009) |
| Student | -0.015 | 0.004 | -0.020 | -0.001 |
|  | (0.011) | (0.004) | (0.013) | (0.004) |
| University | -0.010 | -0.003 | 0.003 | -0.000 |
|  | (0.009) | (0.002) | (0.007) | (0.004) |
| Diploma | 0.015 | -0.001 | -0.013 | 0.010 |
|  | (0.015) | (0.005) | (0.020) | (0.006) |
| Certificate 3 or 4 | -0.013 | -0.015 | -0.011 | 0.016 |
|  | (0.034) | (0.011) | (0.042) | (0.013) |
| Year 12 | 0.014 | 0.005 | -0.002 | 0.009 |
|  | (0.019) | (0.006) | (0.021) | (0.007) |
| Married | -0.048^*^ | -0.015^*^ | 0.011 | -0.014^**^ |
|  | (0.027) | (0.009) | (0.030) | (0.007) |
| Defacto | -0.171 | -0.073^*^ | 0.048 | -0.046 |
|  | (0.107) | (0.039) | (0.123) | (0.028) |
| Separated | -0.048 | -0.020 | 0.009 | -0.014 |
|  | (0.037) | (0.013) | (0.041) | (0.010) |
| Divorced | -0.059 | -0.031 | -0.008 | -0.010 |
|  | (0.054) | (0.020) | (0.062) | (0.013) |
| Single | -0.308 | -0.183^*^ | -0.072 | -0.082 |
|  | (0.284) | (0.104) | (0.332) | (0.073) |
| Aus. born | -0.023 | 0.024 | -0.456^**^ | -0.099 |
|  | (0.170) | (0.063) | (0.210) | (0.076) |
| Born main English | -0.009 | 0.003 | -0.042^*^ | -0.014^*^ |
|  | (0.017) | (0.006) | (0.023) | (0.008) |
| Major urban | 0.033 | 0.050 | -0.341 | -0.066 |
|  | (0.196) | (0.092) | (0.255) | (0.121) |
| Other urban | 0.007 | 0.007 | -0.050 | -0.016 |
|  | (0.043) | (0.019) | (0.055) | (0.025) |
| Rural balance | -0.004 | -0.001 | -0.030 | -0.002 |
|  | (0.012) | (0.005) | (0.020) | (0.008) |
| Constant | 3.510^***^ | 1.142^***^ | -1.715^**^ | 0.640^**^ |
|  | (0.725) | (0.259) | (0.823) | (0.250) |
| $n$ | 11612 | 11612 | 11695 | 11673 |
| $n_{jh}$ | 850 | 850 | 853 | 852 |
| $n_{hilda}$ | 1076 | 10762 | 10842 | 10821 |

Notes: The estimates for the HILDA group are weighted by the probability of not being in the severely disadvantaged population (see Appendix S3). See Table C1 for additional details.

Finally, we re-estimate our baseline decomposition models but instead of using $\hat{\beta}_{hilda}$ for the counterfactual in Equation 1 we use $\hat{\beta}_{jh}$ (i.e. we use coefficient weights from the Journeys Home sample for the counterfactual explained gap calculations). In this alternative decomposition, we can fully explain the gaps in wellbeing. The essence of our results is largely unchanged however; psychological resources explain a larger fraction of the gaps than other controls collectively and locus of control and emotional stability continue to have the largest independent effects, while cognition and risk willingness have little explanatory power.

**Fig D2. Gaps in Mental Wellbeing and Decomposition Results – Alternate Counterfactual Coefficients**

Notes: Graphical depiction of estimates in Table D3 (after normalizing the gaps to equal one).

**Table D3: Detailed Blinder-Oaxaca Decomposition Results – Alternate Counterfactual Coefficients**

|  | Mental distress | High mental distress | Life satisfaction | Loneliness |
| --- | --- | --- | --- | --- |
| *Overall* |  |  |  |  |
| Mean (JH) | 0.840^***^ | 0.158^***^ | -0.579^***^ | 0.312^***^ |
|  | (0.068) | (0.017) | (0.074) | (0.022) |
| Mean (HILDA) | -0.066^***^ | 0.037^***^ | 0.021^*^ | 0.169^***^ |
|  | (0.012) | (0.003) | (0.012) | (0.005) |
| Difference | 0.906^***^ | 0.121^***^ | -0.600^***^ | 0.143^***^ |
|  | (0.069) | (0.017) | (0.075) | (0.023) |
| Explained | 0.931^***^ | 0.118^***^ | -0.534^**^ | 0.185^***^ |
|  | (0.153) | (0.043) | (0.210) | (0.053) |
| Unexplained | -0.025 | 0.004 | -0.066 | -0.042 |
|  | (0.143) | (0.041) | (0.204) | (0.050) |
| *Explained* |  |  |  |  |
| Risk willingness | -0.042 | -0.015 | 0.026 | -0.003 |
|  | (0.035) | (0.011) | (0.034) | (0.011) |
| Internal locus of control | 0.205^***^ | 0.037^***^ | -0.200^***^ | 0.057^***^ |
|  | (0.035) | (0.010) | (0.040) | (0.012) |
| Cognition | 0.004 | -0.000 | 0.052^**^ | -0.004 |
|  | (0.018) | (0.006) | (0.022) | (0.007) |
| Emotional stability | 0.056^**^ | 0.007 | -0.016 | 0.017^*^ |
|  | (0.024) | (0.008) | (0.027) | (0.009) |
| Conscientiousness | 0.040^**^ | 0.005 | -0.055^**^ | -0.004 |
|  | (0.020) | (0.006) | (0.025) | (0.007) |
| Social capital | 0.043^**^ | 0.010^**^ | -0.048^**^ | 0.014^**^ |
|  | (0.020) | (0.005) | (0.022) | (0.006) |
| Sleep quality | 0.072^***^ | 0.015^***^ | -0.075^***^ | 0.012^**^ |
|  | (0.020) | (0.005) | (0.021) | (0.005) |
| Long term health cond. | 0.048^***^ | 0.006 | -0.014 | 0.009^*^ |
|  | (0.016) | (0.004) | (0.014) | (0.005) |
| Parents separated age 16 | -0.005 | 0.004 | 0.005 | 0.013 |
|  | (0.035) | (0.011) | (0.042) | (0.012) |
| Parents never together | 0.006 | -0.001 | -0.007 | 0.004 |
|  | (0.008) | (0.003) | (0.014) | (0.004) |
| Mother university degree | -0.003 | 0.000 | 0.001 | 0.000 |
|  | (0.004) | (0.001) | (0.004) | (0.002) |
| Age | 0.181^**^ | 0.028 | 0.037 | -0.013 |
|  | (0.077) | (0.023) | (0.096) | (0.029) |
| Male | -0.008 | -0.001 | -0.031^*^ | 0.006 |
|  | (0.016) | (0.005) | (0.018) | (0.006) |
| ATSI | -0.028^*^ | -0.007 | 0.027 | -0.004 |
|  | (0.016) | (0.004) | (0.023) | (0.006) |
| Student | -0.002 | 0.001 | -0.001 | -0.000 |
|  | (0.002) | (0.001) | (0.002) | (0.001) |
| University | 0.052 | 0.017 | 0.004 | -0.004 |
|  | (0.052) | (0.014) | (0.040) | (0.022) |
| Diploma | -0.002 | 0.000 | 0.003 | -0.001 |
|  | (0.004) | (0.001) | (0.005) | (0.002) |
| Certificate 3 or 4 | -0.003 | -0.004 | -0.010 | 0.006 |
|  | (0.010) | (0.003) | (0.013) | (0.004) |
| Year 12 | -0.007 | -0.002 | 0.002 | -0.005 |
|  | (0.009) | (0.003) | (0.010) | (0.003) |
| Married | 0.605^**^ | 0.187^*^ | -0.141 | 0.210^***^ |
|  | (0.276) | (0.097) | (0.340) | (0.066) |
| Defacto | -0.084 | -0.038^*^ | 0.026 | -0.035^**^ |
|  | (0.057) | (0.021) | (0.063) | (0.016) |
| Separated | -0.026 | -0.012 | -0.010 | -0.009 |
|  | (0.024) | (0.009) | (0.025) | (0.007) |
| Divorced | -0.023 | -0.014 | -0.012 | -0.006 |
|  | (0.026) | (0.010) | (0.028) | (0.006) |
| Single | -0.156 | -0.112^*^ | -0.071 | -0.070 |
|  | (0.182) | (0.067) | (0.213) | (0.046) |
| Aus. born | -0.006 | 0.003 | -0.056^*^ | -0.011 |
|  | (0.024) | (0.009) | (0.032) | (0.011) |
| Born main English | 0.006 | -0.002 | 0.025 | 0.008 |
|  | (0.011) | (0.004) | (0.017) | (0.005) |
| Major urban | 0.002 | 0.004 | -0.030 | -0.004 |
|  | (0.017) | (0.008) | (0.024) | (0.011) |
| Other urban | 0.001 | -0.001 | 0.006 | 0.002 |
|  | (0.007) | (0.003) | (0.010) | (0.004) |
| Rural balance | 0.007 | 0.001 | 0.028 | 0.001 |
|  | (0.012) | (0.005) | (0.020) | (0.008) |
| *Unexplained* |  |  |  |  |
| Risk willingness | -0.090 | -0.033 | 0.057 | -0.020 |
|  | (0.102) | (0.032) | (0.099) | (0.032) |
| Internal locus of control | -1.055^***^ | -0.292^***^ | 1.283^***^ | -0.359^***^ |
|  | (0.289) | (0.100) | (0.363) | (0.115) |
| Cognition | 0.012 | 0.001 | -0.447^**^ | 0.025 |
|  | (0.184) | (0.060) | (0.212) | (0.068) |
| Emotional stability | 0.272 | 0.007 | -0.066 | -0.029 |
|  | (0.184) | (0.061) | (0.216) | (0.070) |
| Conscientiousness | -0.119 | -0.009 | 0.504 | 0.052 |
|  | (0.247) | (0.079) | (0.307) | (0.088) |
| Social capital | -1.311^***^ | -0.397^***^ | 1.206^***^ | 0.017 |
|  | (0.306) | (0.109) | (0.392) | (0.127) |
| Sleep quality | -0.200 | -0.109^*^ | 0.412^**^ | -0.093 |
|  | (0.192) | (0.061) | (0.201) | (0.063) |
| Long term health cond. | 0.066^**^ | 0.007 | 0.014 | 0.015 |
|  | (0.030) | (0.009) | (0.035) | (0.011) |
| Parents separated age 16 | -0.007 | 0.001 | 0.006 | 0.004 |
|  | (0.016) | (0.005) | (0.020) | (0.006) |
| Parents never together | 0.002 | 0.000 | -0.001 | 0.001 |
|  | (0.002) | (0.001) | (0.003) | (0.001) |
| Mother university degree | 0.010 | -0.001 | -0.002 | 0.001 |
|  | (0.017) | (0.006) | (0.015) | (0.007) |
| Age | -0.211 | -0.048 | -0.467 | 0.064 |
|  | (0.248) | (0.075) | (0.308) | (0.095) |
| Male | 0.028 | 0.003 | -0.074 | 0.033^*^ |
|  | (0.051) | (0.016) | (0.056) | (0.019) |
| ATSI | -0.007^**^ | -0.001^*^ | 0.003 | -0.001 |
|  | (0.003) | (0.001) | (0.004) | (0.001) |
| Student | -0.013 | 0.003 | -0.017 | -0.001 |
|  | (0.009) | (0.003) | (0.010) | (0.004) |
| University | -0.069 | -0.019 | 0.022 | -0.001 |
|  | (0.061) | (0.016) | (0.048) | (0.025) |
| Diploma | 0.017 | -0.001 | -0.014 | 0.011 |
|  | (0.017) | (0.005) | (0.022) | (0.007) |
| Certificate 3 or 4 | -0.009 | -0.010 | -0.008 | 0.011 |
|  | (0.023) | (0.007) | (0.029) | (0.009) |
| Year 12 | 0.021 | 0.008 | -0.003 | 0.014 |
|  | (0.027) | (0.009) | (0.031) | (0.010) |
| Married | -0.617^**^ | -0.195^*^ | 0.134 | -0.173^**^ |
|  | (0.300) | (0.105) | (0.370) | (0.073) |
| Defacto | -0.082 | -0.035^*^ | 0.023 | -0.022^*^ |
|  | (0.051) | (0.019) | (0.059) | (0.013) |
| Separated | -0.018 | -0.007 | 0.003 | -0.005 |
|  | (0.013) | (0.005) | (0.015) | (0.004) |
| Divorced | -0.032 | -0.017 | -0.004 | -0.006 |
|  | (0.029) | (0.011) | (0.033) | (0.007) |
| Single | -0.110 | -0.065^*^ | -0.025 | -0.029 |
|  | (0.101) | (0.037) | (0.118) | (0.026) |
| Aus. born | -0.019 | 0.020 | -0.388^**^ | -0.085 |
|  | (0.145) | (0.054) | (0.178) | (0.064) |
| Born main English | -0.015 | 0.004 | -0.070^*^ | -0.024^*^ |
|  | (0.028) | (0.010) | (0.037) | (0.012) |
| Major urban | 0.034 | 0.046 | -0.312 | -0.061 |
|  | (0.179) | (0.084) | (0.232) | (0.110) |
| Other urban | 0.009 | 0.008 | -0.059 | -0.019 |
|  | (0.050) | (0.022) | (0.064) | (0.029) |
| Rural balance | -0.008 | -0.001 | -0.061 | -0.004 |
|  | (0.024) | (0.011) | (0.038) | (0.015) |
| Constant | 3.495^***^ | 1.136^***^ | -1.715^**^ | 0.641^**^ |
|  | (0.726) | (0.259) | (0.823) | (0.250) |
| $n$ | 11612 | 11612 | 11695 | 11673 |
| $n_{jh}$ | 850 | 850 | 853 | 852 |
| $n_{hilda}$ | 10762 | 10762 | 10842 | 10821 |

Notes: Estimates use $\hat{\beta}_{jh}$in Eq. S6 (rather than $\hat{\beta}_{hilda}$ as in Table D1). See Table D1 for additional details.

**Appendix S4 References:**

Jann B. The Blinder-Oaxaca decomposition for linear regression models. Stata Journal. 2008;8(4):453–479.
