# Supplementary material for: Psychological, social and cognitive resources and the mental wellbeing of the poor
Source: PLoS One. 2021 Oct 12;16(10):e0258417. doi: 10.1371/journal.pone.0258417 (PMC8509876; doi:10.1371/journal.pone.0258417)
Supplement: S1 Replication files — (ZIP) [file pone.0258417.s002.zip › Replication for PlosOne/readme.docx]

**Data availability**: This study uses data from the Journeys Home Survey and the Household, Income and Labour Dynamics in Australia (HILDA) Survey. Data from the Journeys Home Survey is available for purchase under individual licensing arrangements with the Australian Government’s Department of Social Services. For instructions on how to access the data, email JH@dss.gov.au. Further information is available at https://melbourneinstitute.unimelb.edu.au/journeys-home/for-researchers. Our study can be replicated using any of the data releases, including the International Release (we used the Limited Release). HILDA can be applied for at no cost to researchers through the National Centre for Longitudinal Data Dataverse. Instructions are available at https://dataverse.ada.edu.au/dataverse/hilda. We used the General Release 16; later releases should provide nearly identical results.

Our Stata do files specify where the user needs to insert the path directory to either HILDA or Journeys Home. For HILDA, the path should be to the combined household files (this is always defined in a global at the beginning).

| **Main Stata do files** | |
| --- | --- |
| compare & oaxaca – all.do | This is the main do file. It produces distribution graphs for the various psychological resources and conducts the baseline Blinder-Oaxaca decompositions (with associated figure).  This do file embeds the subsidiary do files for each psychological resource described below. It assumes these do files are saved in the same folder. |
| compare & oaxaca - all weighted.do | This runs the Blinder-Oaxaca decompositions (with associated figure) using the scaled binomial loss model weights. It also produces a figure comparing binary logit to weighted logit, shown in the supplementary material. |
| compare & oaxaca - all reversed.do | This runs the Blinder-Oaxaca decompositions (with associated figure) using the coefficient weights from the Journeys Home group to calculate the explained part. |
| **Subsidiary do files** | |
| compare & oaxaca - big 5.do | This creates a combined Journeys Home and HILDA dataset for comparing emotional stability and conscientiousness. It assumes the relevant Journeys Home and HILDA source files are saved in the same folder. |
| compare & oaxaca – cognition.do | As above, for cognition. |
| compare & oaxaca – loc.do | As above, for internal locus of control. |
| compare & oaxaca - risk preferences.do | As above, for willingness to take risks. |
| compare & oaxaca – sleep.do | As above, for sleep quality. |
| compare & oaxaca - social capital.do | As above, for social capital. |
| compare & oaxaca - mental health.do | As above, for the Kessler 10, life satisfaction and loneliness variables. Because decompositions for these variables use the other psychological resources, this do file also imports those resources from the relevant waves they were collected in. |
| **Other do files** | |
| descriptives table.do | Creates a table of descriptive statistics that appears in the main part of the paper. |
| graph response rate.do | Creates a figure for the response rate across waves in Journeys Home. |
| **Ado files** | |
| o_alpha.ado | A program for calculating ordinal alpha. To use this just type “o_alpha `varlist’” into the command window. It requires the user to install the Stata command *polychoric*. |
| s_bloss_run.ado | A program used to estimate the scaled binomial loss model (needed for “compare & oaxaca - all weighted.do”). |
