# Supplementary material for: Psychological, social and cognitive resources and the mental wellbeing of the poor
Source: PLoS One. 2021 Oct 12;16(10):e0258417. doi: 10.1371/journal.pone.0258417 (PMC8509876; doi:10.1371/journal.pone.0258417)
Supplement: Attachment — Submitted filename: Response to reviewers.pdf [file pone.0258417.s004.pdf]

Response to reviewers

Thank you to both reviewers for your thoughtful comments on our paper. There were many great suggestions, and we believe the paper has improved markedly as a result. Below we provide a detailed response to every comment.

<u>Reviewer 1</u>

1. Axe Section 4.2 Its a re-run of Figure 3 that adds little and suffers from the problem that there are only 28 full homeless in the data.

We accept that the results in Section 4.2 are limited by the small sample sizes of the groups (especially the 'homeless' category) and have therefore removed this section. To accommodate this change, it was also necessary to fold Section 4.1 into Section 3. Results from that section now appear immediately after Figure 1 in the paper.

2. Simplify Section 4.1 to show the average differences in important characteristics (pick your favourite 5 to 10) across the different degrees. Also calculate the implied distance between the 4 categories (ie how many times is the difference in a characteristic between secure 'non-trad' versus 'secure trad' the difference between 'non-secure' and 'secure traditional'). That gives the reader a sense of what the special group is and how different the groups really are.

The cornerstone of the previous Section 4.1 is our figure showing mean differences in resources across different categories of homelessness. We recognize that the previous iteration of this figure did not allow for an easy comparison of relative effect sizes across the different groups. We have therefore put all the estimates into a single figure, which allows for a straightforward comparison of the resources by homelessness status (see below). This figure shows that, for example, the effect sizes are particularly large for locus of control among the insecure and homeless groups.

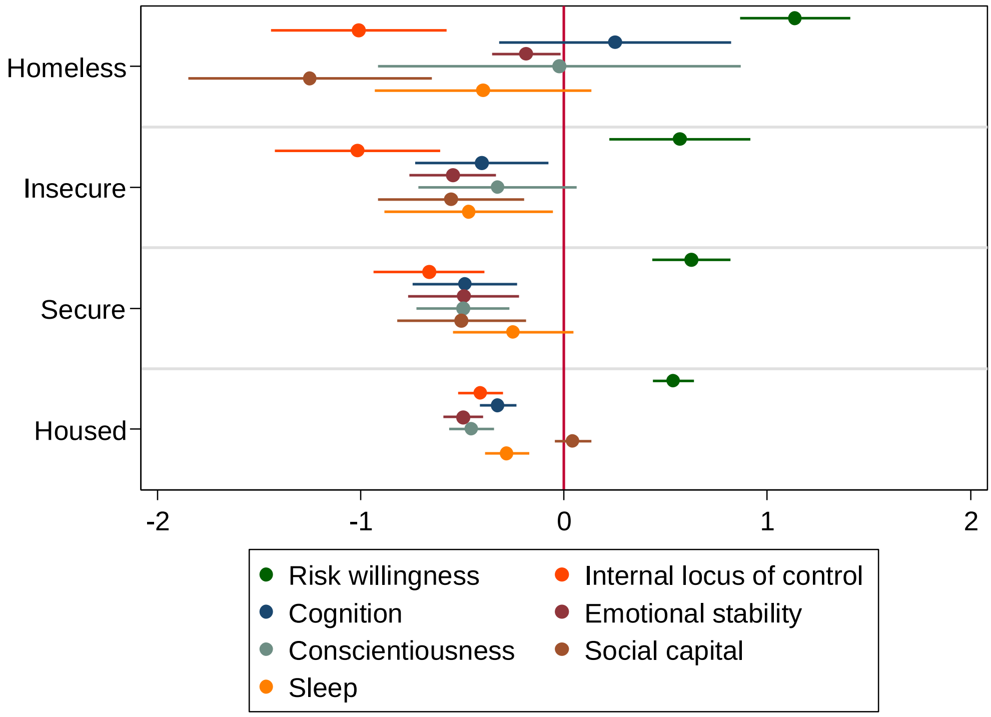

3. Find a way to condense the four figures in Figure 3 to one graph/bar. At present it looks clunky and not memorable. You are trying to sell the Oaxaca decomposition here so make it memorable.

We can understand the suggestion to condense this, but we have four different dependent variables which we believe are each interesting in their own right. Any effort to combine them would involve a loss of important information. While it is evident that the bars are all quite similar in terms of the key predictors, this was not known a priori, and having the four bars makes this important discovery clear to a reader.

With that said, we believe it is possible to improve the clarity of this figure without loss of information. To that end, we have revised the figure by rescaling the y-axis on each chart such that the unconditional gaps are equal to one, and placing each bar within a single chart. We then report the absolute figures for supplementary material. This allows the reader to compare across the different dependent variables on the same scale and side-by-side.

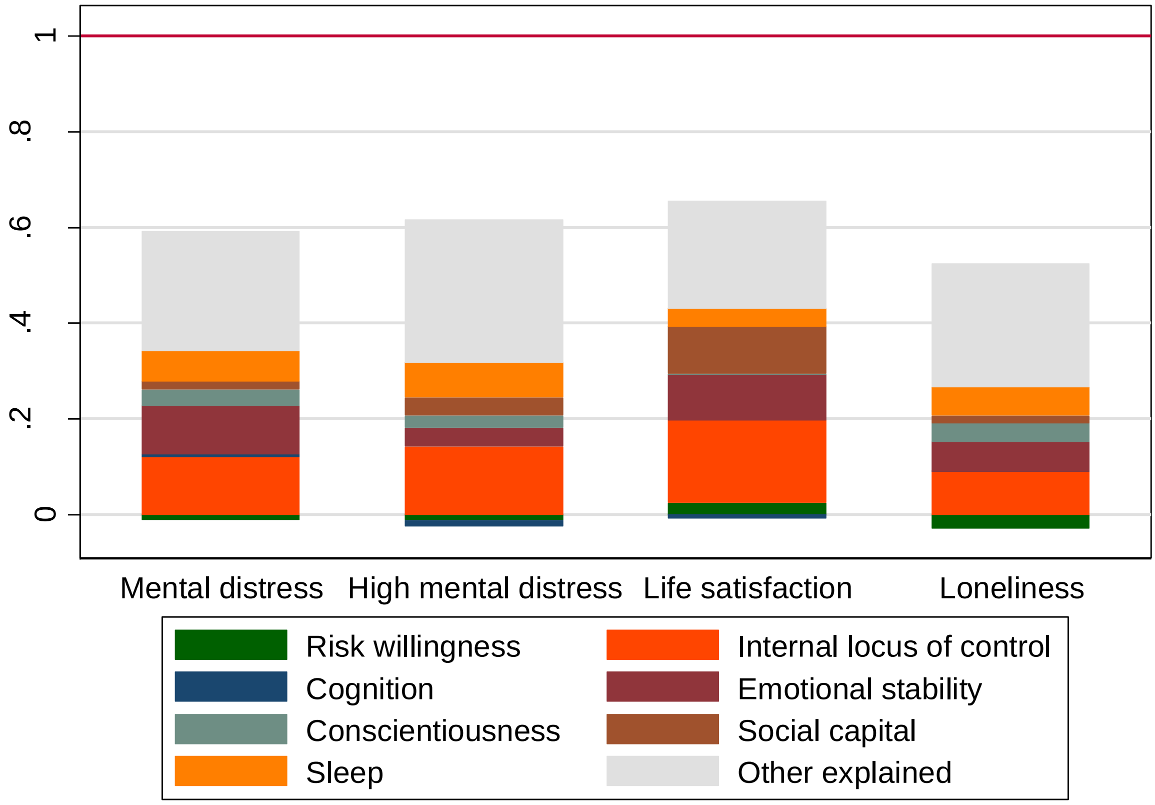

4. Change the labeling of the categories of variables. The word 'resources' is a very poor descriptor because it includes items that have for decades been called different things. Emotional stability and conscientiousness are two of the big-5 personality traits. Sleep is part of the GHQ12 notion of mental wellbeing itself (rather than a resource into it). I would just call them psychological characteristics and outcomes. Similarly, avoid causal language since many items put on the right-hand side could be on the left-hand side (so Figure 3 is a big stretch: why sleep is an input rather than an output is arbitrary).

We have given this careful consideration and decided to retain the use of the word resources. We believe that "resources" is, in fact, a very apt descriptor for the broad concept we have in mind—i.e., the qualities, skills, and support systems that assist people in achieving their goals and dealing with problems effectively. (For common English definitions of "resources" see https://www.macmillandictionary.com/dictionary/british/resource_1). Moreover, it is a term that is often used in the literature to explain why people living in disadvantaged circumstances are more susceptible to the adverse consequences of stress (e.g. Taylor and Seeman 1999; Matthews et al. 2010).

Our objective is not to relabel existing concepts. Instead, we have used "resources" as an umbrella term to capture a wide range of existing concepts across psychology (Big Five personality traits, locus of control); economics (risk preferences); sociology (notions of social capital); and cognitive sciences (cognition). Nor do we wish to take a stand on causality. We are aware, for example, that there is a complex relationship between mental health, cognition and sleep making the direction of causality difficult to isolate. Rather our goal is to document the disparity in the resources that very vulnerable people have at their disposal.

We have responded to this comment in two ways. First, we have added a justification for our use of the word “resources” in online Appendix S1 (Section A.4) where we describe the main variables included in our analysis. This new discussion immediately proceeds Table A.1 which links the original concept to the measures in our two data sources. Second, we have carefully edited the paper to remove any inadvertent causal language.

Taylor SE, Seeman TE. Psychosocial resources and the SES-health relationship. Ann N Y Acad Sci. 1999;896:210–25.

Matthews KA, Gallo LC, Taylor SE. Are psychosocial factors mediators of socioeconomic status and health connections? A progress report and blueprint for the future. Ann N Y Acad Sci. 2010;1186:146–173.

5. Condense the literature sections. The reader mainly needs to know whether your descriptives are truly new: was it already known how much the homeless differ from the rest of the population in all those psychological dimensions?

The comments of both reviewers have made it clear to us that our initial manuscript did not go far enough in interpreting our results and placing them in the context of the previous studies. We have undertaken a major rewrite of the paper to rectify this.

Specifically, we have responded to this comment (and the one following) in several ways. First, we have restructured the paper, shortening Section 1 (Introduction) and removing some details of the previous literature. Second, we have expanded Section 4 (Discussion), focusing it directly on what our results add to previous studies. In making these changes, we were mindful of the need to be concise and the suggestion from the referee to condense our literature sections.

See also our response to Referee 2 (comments #1 and #2) who makes a related point in asking for a more detailed analysis of the policy implications of our results.

6. I hence like the degree to which the paper informs me about the differences between the homeless and the rest, but I need to be told that this papers adds to what is known about that difference, and the paper needs to be shortened and sharpened to stick to those descriptives.

By carefully rewriting and restructuring the paper (as described above), we were able to sharpen the focus on what can be learned from the analysis that we have done. We believe that the paper has improved considerably as a result.

Reviewer 2

1. The findings are backed with sound methodology and sufficient sample size. And this is a positive aspect of the study. However, the research's significance and values are not addressed adequately in the text. The discussion lacks depth and needs further results' clarification with existing policies regarding homelessness. The rising complexities of the problem have become far more difficult to measure than simplistic results we can see from a theoretical model or two.

The comments of both reviewers have made it clear to us that our initial manuscript did not go far enough in interpreting our results and placing them in the context of the previous studies. We have undertaken a major rewrite of the paper to rectify this.

Specifically, we have responded to this comment by expanding our discussion (Section 4) of how our results inform policy options targeting housing insecurity. We now say:

> **Our focus on examining poverty through the lens of housing vulnerability is novel. In their recent review, Cox et al. (2019) note that there has been a strong research emphasis on homelessness, arguing that the failure to take a broader perspective and consider all aspects of housing insecurity (e.g. housing affordability, stability, safety, etc.) has "resulted in much less being known about its true prevalence and the actual costs it imposes on society" (p. 95). Our results contribute to closing this gap by quantifying the large degree to which housing vulnerability is associated with increased mental distress and loneliness as well as a reduction in life satisfaction. We show that people's personal resources matter as much, or more, than their demographic characteristics and family background combined in driving the impact that housing vulnerability has on their diminished mental wellbeing.**

See also our response to Referee 1 (comments # 5 and #6) who makes a similar point about the need to interpret our results and place them in context.

2. I think some critical aspects should not be left out when we discuss the health issues of vulnerable people. Specifically: healthcare disparity https://ajph.aphapublications.org/doi/abs/10.2105/AJPH.2013.301490, https://springerplus.springeropen.com/articles/10.1186/s40064-015-1279-x), health care system (https://ajph.aphapublications.org/doi/full/10.2105/AJPH.2005.076190), and healthcare discrimination (https://bmchealthservres.biomedcentral.com/articles/10.1186/1472-6963-14-376), to be specific.

While there are numerous policy challenges stemming from housing insecurity, we agree with the Referee that health issues are at the forefront. We have expanded the discussion in Section 4 to consider the implications of our results for the health issues faced by people who lack secure housing.

In particular, we now say:

> **It is also the case that, housing insecurity is related both to unhealthy behaviors and poor health outcomes (Stahre et al. 2011). Moreover, in wealthy countries, like Australia, where social and economic conditions are more favorable fewer people are likely to lack secure housing, while those who do may be particularly disadvantaged relative to the rest of society (see Shinn 2007; Milburn et al. 2007). Policies targeting the health challenges stemming from housing vulnerability – or indeed disadvantage more generally – need to be cognizant of the constraints imposed by people's personality traits, risk preferences, control perceptions, cognition, and social capital.**

3. As the authors stated, "The way that people perceive the world, process information, and make decisions is shaped by their psychological, social, and cognitive resources" I advise the authors to take a look at the Mindsponge mechanism (https://www.sciencedirect.com/science/article/abs/pii/S0147176715000826; https://www.taylorfrancis.com/chapters/global-mindset-integration-emerging-sociocultural-values-mindsponge-processes-quan-hoang-vuong/e/10.4324/9781315736396-8), which might provide theoretical support for the study's objectives.

We thank the reviewer for drawing our attention to this interesting work on people's mindset. Unfortunately, although our data are extremely detailed allowing us to explore a broad range of psycho-social and cognitive concepts in a single analysis, the reality is that we cannot measure everything that we might be interested in. We have no empirical measure of people's mindset, cultural values, resilience, etc. which limits the usefulness of these constructs as the theoretical basis for our study.

Instead, we have broadened this statement to recognize that people's psychological, social and cognitive resources do not operate independently of people's beliefs and cultural values. Specifically, on p. 1 we now say:

> **The way that people perceive the world, process information, and make decisions is shaped not only by their beliefs and values, but also by their psychological, social, and cognitive resources.**

4. Figure 1's stacked bar charts should be replaced by clustered bar charts for better clarity.

We have modified the figure as suggested (reproduced below).

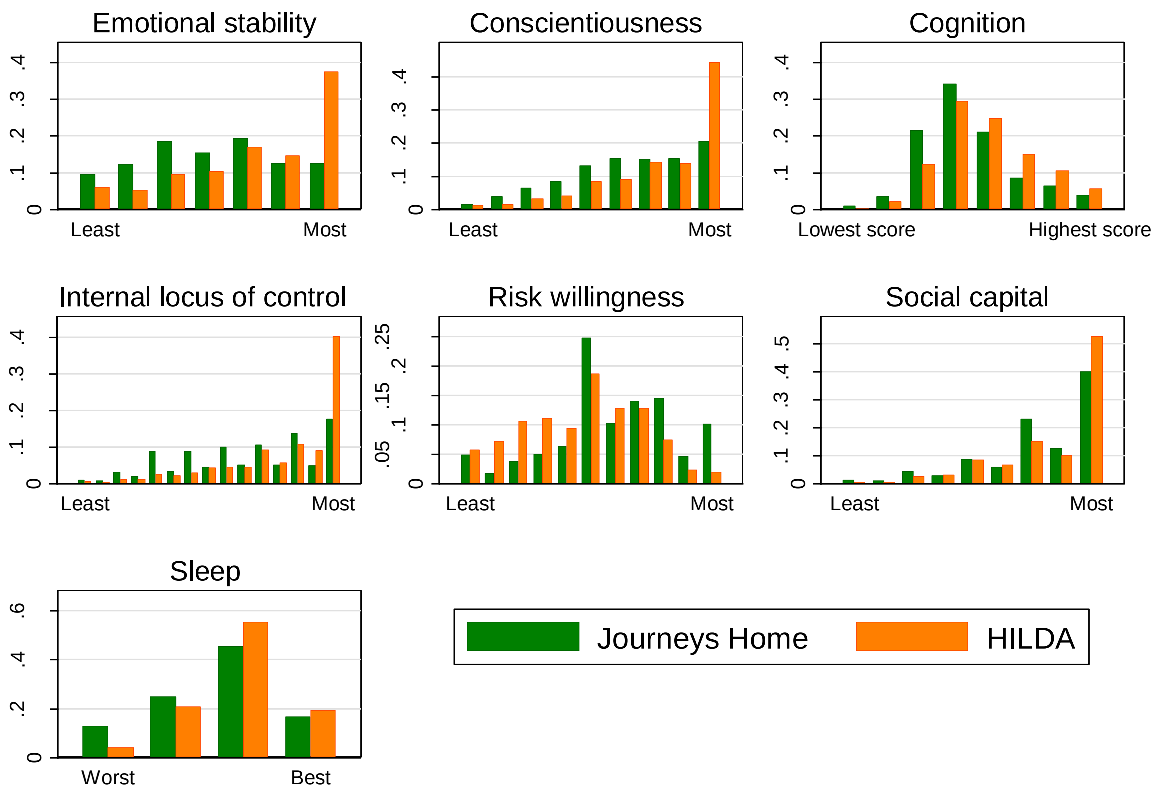

5. The paper's structure needs to be revised. Model 1 should be placed in the corresponding section where the main methodological discussions were provided. Suppose the authors want to present mathematical models together with the results. In that case, I recommend the authors clearly address all the components of the model rather than keeping the general form (like Model 1).

On reflection, we realize that specifying an equation for model 1 is distracting and unnecessary given we are estimating a simple logit regression, which is a well-known model. We have therefore removed this equation from the paper and describe our estimation in the text.

6. Please specify the high-income country.

We have made this change to the Abstract and Introduction.

7. Last but not least, the study's limitations are needed, please refer to this article for modern standards: https://www.nature.com/articles/d41586-020-01694-x

We have added a paragraph to the discussion section explaining the main limitations of our study.

> **In reaching these conclusions, we are cognizant of the limitations of our study. We recognize that the relationships between homelessness, resources and mental wellbeing are likely complex and bi-directional. While our study provides**

important descriptive evidence, given the observational nature of our data, our estimates cannot be interpreted as causal. We also acknowledge limitations in the comparison of resources across datasets owing to some differences in wording, scales and timing of questionnaires (see Table A1 in S1 Appendix), although there is no reason to think these differences should influence results in the direction of our conclusions.
