# Supplementary material for: Psychological, social and cognitive resources and the mental wellbeing of the poor
Source: PLoS One. 2021 Oct 12;16(10):e0258417. doi: 10.1371/journal.pone.0258417 (PMC8509876; doi:10.1371/journal.pone.0258417)
Supplement: Attachment — Submitted filename: response letter.pdf [file pone.0258417.s005.pdf]

Nathan Kettlewell

The University of Technology Sydney
Ultimo, Sydney, NSW 2007

Abid Hussain
PLOS ONE

---

12 August 2021

Dear Professor Hussain,

We are pleased that our edits were generally well received and appreciate the further comments from Reviewer #1.

Issue:

The only remaining concern is around our use of the term ‘resources’ when describing the overall contribution of the seven factors we consider (risk, locus of control, emotional stability, cognition, sleep, social capital) to our four mental wellbeing measures. The reviewer has no issue with how we define psychosocial and cognitive resources, but takes exception to sleep and risk orientation being included in our group of `resources’.

Our Response:

We completely accept the argument that sleep and risk (as well as other resources we study), are not only constructs that can be drawn upon to assist with life goals, but are also likely to be outcomes of personal circumstances. This bi-directional causality is something we acknowledge throughout the paper (see especially Section 5) and is why our results are descriptive.

However, we disagree that sleep and risk orientation do not meet our definition of a resource. We are not alone in viewing sleep as a resource (see e.g., Hamilton et al 2007; Barnes & Spreitzer 2015). Sleep deprivation can lead to cognitive impairment, reduced effort and anti-social behaviors. A person’s stock of quality sleep is therefore something that can be drawn on as a protective factor. Similarly, while risk orientation may well be influenced by poverty, it is also a trait that can support decision making.

Ultimately, reasonable people will disagree on where to draw the line on what is a resource and what is not. Our approach is to be as transparent as possible by clearly stating our reasoning for viewing these seven factors as resources and referencing the relevant literature. We explicitly list each (risk, locus of control, contentiousness, emotional stability, cognition, sleep, social capital) in the abstract and elsewhere in the paper to make our use of the term `resources’ clear.

Importantly, in Table D1 (reproduced below), we report the detailed decomposition results. If a reader – like the referee – wishes to exclude any particular set of resources and recalculate the ‘headline figure’, this is easy to do. In practice, excluding sleep and risk has a modest effect

on the headline figure because these resources explain a relatively small fraction of the wellbeing deficit between the housing insecure and general population groups (see Figure 5). The figure we report in the paper is 24-42% – excluding sleep and risk, this would be 21-36%.

In response to the referee's comment and to improve transparency, we have revised the paper as follows:

First, we now cite studies that discuss sleep as a resource on page 8. Second, on page 13 we now direct readers to Table D1 for the detailed decomposition estimates, which show "the individual contributions of each resource and other controls" so that they are able to construct the `headline figure' however they like.

We look forward to hearing from you.

Sincerely,

Nathan Kettlewell

Table D1: Detailed Blinder-Oaxaca Decomposition Results – Baseline

	Mental distress	High mental distress	Life satisfaction	Loneliness
Overall				
Mean (JH)	0.840***	0.158***	-0.579***	0.312***
	(0.068)	(0.017)	(0.074)	(0.022)
Mean (HILDA)	-0.066***	0.037***	0.021*	0.169***
	(0.012)	(0.003)	(0.012)	(0.005)
Difference	0.906***	0.121***	-0.600***	0.143***
	(0.069)	(0.017)	(0.075)	(0.023)
Explained	0.525***	0.060***	-0.355***	0.092***
	(0.037)	(0.007)	(0.034)	(0.013)
Unexplained	0.382***	0.061***	-0.245***	0.051**
	(0.057)	(0.018)	(0.068)	(0.021)
Explained				
Risk willingness	-0.011	-0.004*	0.007	0.003
	(0.007)	(0.002)	(0.008)	(0.003)
Internal locus of control	0.109***	0.011***	-0.085***	0.025***
	(0.014)	(0.002)	(0.012)	(0.004)
Cognition	0.005	0.000	0.008**	-0.001
	(0.003)	(0.001)	(0.004)	(0.001)
Emotional stability	0.091***	0.008***	-0.024***	0.014***
	(0.011)	(0.002)	(0.006)	(0.003)
Conscientiousness	0.030***	0.005***	-0.015***	0.000
	(0.006)	(0.002)	(0.005)	(0.002)
Social capital	0.016**	0.002**	-0.023**	0.014**
	(0.007)	(0.001)	(0.010)	(0.006)
Sleep quality	0.057***	0.007***	-0.044***	0.005***
	(0.012)	(0.002)	(0.009)	(0.002)
Long term health cond.	0.022***	0.003***	-0.019***	0.003**
	(0.005)	(0.001)	(0.005)	(0.001)
Parents separated age 16	0.009	0.002	-0.008	0.005
	(0.008)	(0.002)	(0.009)	(0.004)
Parents never together	-0.005	-0.001	-0.003	0.001
	(0.005)	(0.001)	(0.007)	(0.003)
Mother university degree	-0.001	-0.000	0.000	0.000
	(0.001)	(0.000)	(0.001)	(0.000)
Age	0.115***	0.013***	-0.109***	0.007
	(0.012)	(0.003)	(0.013)	(0.005)
Male	-0.016***	-0.002**	-0.008**	-0.005***
	(0.004)	(0.001)	(0.004)	(0.002)
ATSI	0.016*	0.003	0.009	0.003
	(0.009)	(0.003)	(0.011)	(0.005)
Student	0.001	-0.000	0.002	-0.000
	(0.001)	(0.000)	(0.003)	(0.000)
University	-0.007	0.001	0.023**	-0.005*
	(0.006)	(0.002)	(0.009)	(0.003)
Diploma	-0.000	-0.000	0.001	-0.000
	(0.001)	(0.000)	(0.002)	(0.000)
Certificate 3 or 4	0.001	0.000	-0.006*	0.000

	(0.003)	(0.001)	(0.004)	(0.001)
Year 12	-0.001	0.000	0.001	-0.000
	(0.002)	(0.000)	(0.002)	(0.001)
Married	0.037*	0.008	-0.018	0.051***
	(0.021)	(0.006)	(0.028)	(0.012)
Defacto	0.004	-0.000	0.001	-0.011***
	(0.005)	(0.001)	(0.006)	(0.003)
Separated	0.005	0.001	-0.016***	-0.001
	(0.004)	(0.001)	(0.006)	(0.002)
Divorced	0.004	0.000	-0.009**	-0.001
	(0.003)	(0.001)	(0.004)	(0.001)
Single	0.043**	0.006	-0.025	-0.017*
	(0.020)	(0.006)	(0.023)	(0.010)
Aus. born	-0.003	-0.000	0.012**	0.004*
	(0.005)	(0.002)	(0.006)	(0.002)
Born main English	0.000	-0.000	-0.004	-0.002*
	(0.002)	(0.001)	(0.003)	(0.001)
Major urban	-0.002	-0.000	0.000	0.002
	(0.003)	(0.001)	(0.004)	(0.002)
Other urban	0.002	0.000	-0.002	-0.001
	(0.002)	(0.000)	(0.002)	(0.001)
Rural balance	0.003	0.000	-0.003	-0.001
	(0.002)	(0.001)	(0.003)	(0.001)
Unexplained				
Risk willingness	-0.121	-0.045	0.076	-0.026
	(0.137)	(0.044)	(0.134)	(0.043)
Internal locus of control	-0.959***	-0.266***	1.167***	-0.327***
	(0.263)	(0.091)	(0.330)	(0.104)
Cognition	0.011	0.001	-0.403**	0.022
	(0.165)	(0.054)	(0.191)	(0.061)
Emotional stability	0.237	0.006	-0.058	-0.025
	(0.161)	(0.053)	(0.189)	(0.061)
Conscientiousness	-0.110	-0.008	0.464	0.048
	(0.227)	(0.073)	(0.283)	(0.081)
Social capital	-1.284***	-0.389***	1.181***	0.017
	(0.300)	(0.106)	(0.384)	(0.125)
Sleep quality	-0.185	-0.101*	0.381**	-0.086
	(0.178)	(0.056)	(0.186)	(0.059)
Long term health cond.	0.093**	0.010	0.019	0.021
	(0.042)	(0.013)	(0.049)	(0.016)
Parents separated age 16	-0.022	0.004	0.020	0.012
	(0.052)	(0.016)	(0.063)	(0.018)
Parents never together	0.013	0.001	-0.005	0.003
	(0.011)	(0.004)	(0.018)	(0.006)
Mother university degree	0.008	-0.001	-0.002	0.001
	(0.013)	(0.005)	(0.012)	(0.005)
Age	-0.145	-0.033	-0.321	0.044
	(0.170)	(0.052)	(0.212)	(0.065)
Male	0.037	0.004	-0.097	0.043*
	(0.067)	(0.021)	(0.074)	(0.025)

ATSI	-0.051**	-0.011*	0.021	-0.008
	(0.021)	(0.006)	(0.029)	(0.009)
Student	-0.015	0.004	-0.020	-0.001
	(0.011)	(0.004)	(0.013)	(0.004)
University	-0.010	-0.003	0.003	-0.000
	(0.009)	(0.002)	(0.007)	(0.004)
Diploma	0.015	-0.001	-0.013	0.010
	(0.015)	(0.005)	(0.020)	(0.006)
Certificate 3 or 4	-0.013	-0.015	-0.011	0.016
	(0.034)	(0.011)	(0.042)	(0.013)
Year 12	0.014	0.005	-0.002	0.009
	(0.019)	(0.006)	(0.021)	(0.007)
Married	-0.048*	-0.015*	0.011	-0.014**
	(0.027)	(0.009)	(0.030)	(0.007)
Defacto	-0.171	-0.073*	0.048	-0.046
	(0.107)	(0.039)	(0.123)	(0.028)
Separated	-0.048	-0.020	0.009	-0.014
	(0.037)	(0.013)	(0.041)	(0.010)
Divorced	-0.059	-0.031	-0.008	-0.010
	(0.054)	(0.020)	(0.062)	(0.013)
Single	-0.308	-0.183*	-0.072	-0.082
	(0.284)	(0.104)	(0.332)	(0.073)
Aus. born	-0.023	0.024	-0.456**	-0.099
	(0.170)	(0.063)	(0.210)	(0.076)
Born main English	-0.009	0.003	-0.042*	-0.014*
	(0.017)	(0.006)	(0.023)	(0.008)
Major urban	0.037	0.051	-0.342	-0.067
	(0.196)	(0.092)	(0.255)	(0.121)
Other urban	0.008	0.007	-0.050	-0.016
	(0.043)	(0.019)	(0.055)	(0.025)
Rural balance	-0.004	-0.001	-0.030	-0.002
	(0.012)	(0.005)	(0.020)	(0.008)
Constant	3.495***	1.136***	-1.715**	0.641**
	(0.726)	(0.259)	(0.823)	(0.250)
n	11612	11612	11695	11673
n_{jh}	850	850	853	852
n_{hilda}	10762	10762	10842	10821

Notes: See Section 3.2 for formulaic details. Estimates were obtained using the -oaxaca- command for Stata (Jann 2008). See Table A1 for further details on the dependent variables and psychological resource variables and Table A2 for further details on the other controls. Asymptotic standard errors in parentheses. * $p < 0.1$, ** $p < 0.05$, *** $p < 0.01$.
