# Supplementary material for: Psychological, social and cognitive resources and the mental wellbeing of the poor
Source: PLoS One. 2021 Oct 12;16(10):e0258417. doi: 10.1371/journal.pone.0258417 (PMC8509876; doi:10.1371/journal.pone.0258417)
Supplement: Attachment — Submitted filename: Review.docx [file pone.0258417.s003.docx]

**Review Report**

Date: March 16, 2021

Manuscript titled: Psychological, Social and Cognitive Resources and the Mental Wellbeing of the Poor

*PLOS One*

My general evaluation: the manuscript has been well prepared, with its methodological section being crafted sufficiently.

The study provides valuable insights that might contribute to policymaking towards homeless people in Australia. Therefore, I trust that the paper has merit to become a contribution to the literature. Nonetheless, I have some issues with the paper and believe they should be addressed before the paper can be ready for publication.

Please find below my specific comments, which might enable the authors to strengthen the paper and its flow of logic.

The findings are backed with sound methodology and sufficient sample size. And this is a positive aspect of the study. However, the research’s significance and values are not addressed adequately in the text.

The discussion lacks depth and needs further results’ clarification with existing policies regarding homelessness. The rising complexities of the problem have become far more difficult to measure than simplistic results we can see from a theoretical model or two.

I think some critical aspects should not be left out when we discuss the health issues of vulnerable people. Specifically: healthcare disparity (<https://ajph.aphapublications.org/doi/abs/10.2105/AJPH.2013.301490>, <https://springerplus.springeropen.com/articles/10.1186/s40064-015-1279-x>), health care system (<https://ajph.aphapublications.org/doi/full/10.2105/AJPH.2005.076190>), and healthcare discrimination (<https://bmchealthservres.biomedcentral.com/articles/10.1186/1472-6963-14-376>), to be specific.

As the authors stated, “The way that people perceive the world, process information, and make decisions is shaped by their psychological, social, and cognitive resources” I advise the authors to take a look at the Mindsponge mechanism (<https://www.sciencedirect.com/science/article/abs/pii/S0147176715000826>; <https://www.taylorfrancis.com/chapters/global-mindset-integration-emerging-socio-cultural-values-mindsponge-processes-quan-hoang-vuong/e/10.4324/9781315736396-8>), which might provide theoretical support for the study’s objectives.

Figure 1’s stacked bar charts should be replaced by clustered bar charts for better clarity

The paper’s structure needs to be revised. Model 1 should be placed in the corresponding section where the main methodological discussions were provided.

Suppose the authors want to present mathematical models together with the results. In that case, I recommend the authors clearly address all the components of the model rather than keeping the general form (like Model 1).

Please specify the high-income country.

Last but not least, the study’s limitations are needed, please refer to this article for modern standards: https://www.nature.com/articles/d41586-020-01694-x
